# Reduced low-prevalence visual search detriment with increasing age: Implications for cognitive theories of aging and real-world search tasks

**DOI:** 10.3758/s13423-024-02457-9

**Published:** 2024-01-30

**Authors:** Stephanie C. Goodhew, Mark Edwards

**Affiliations:** School of Medicine and Psychology, Psychology Building (building 39), The Australian National University, Canberra, ACT 2601 Australia

**Keywords:** Visual search, Low prevalence, Aging, Processing speed, Compensation, Quitting threshold

## Abstract

When performing multiple successive visual searches, low-prevalence targets are at elevated risk of being missed. This has important implications for real-world visual search tasks, such as diagnostic medical imaging (e.g., searching for a cancer) and airport baggage security screening (e.g., searching for a weapon), which are characterized by low-prevalence targets and potentially dire consequences of target misses. Previous work on low-prevalence visual search indicates that individuals who spontaneously respond more slowly miss fewer targets, which has been attributed to higher quitting thresholds predicting better performance. Previous aging research indicates that older adults typically respond more slowly across multiple task contexts. This has been attributed to both intrinsic limitations in processing speed and a strategic trade-off to prioritize accuracy with increasing age. Synthesizing these two separate lines of research, here we tested whether older adults had a higher quitting threshold and/or slower processing speed in low-prevalence visual search, and the consequences of these for the magnitude of the low-prevalence visual search detriment. We recruited a large sample (N = 380) across a range of ages (20–80 years) and had them search for targets under low- and high-prevalence conditions. Older adults had both slower processing speed and higher quitting thresholds. Older adults were moderately less susceptible to the low-prevalence detriment, and this relationship was mediated by countervailing effects: slower processing speed exacerbated older adults’ low-prevalence detriment, whereas elevated quitting threshold mitigated it. Theoretical implications for cognitive aging and practical implications for professional visual search tasks are discussed.

**Supplementary Information:**

The online version contains supplementary material available at 10.3758/s13423-024-02457-9.

## Introduction

Visual search is the process of searching for a target amongst other objects, such as scanning a crowd for the face of a friend or searching your office for your keys. Many factors impact the efficiency of visual search (Wolfe, 2021; Wolfe & Horowitz, 2017). However, visual searches can also differ in their effectiveness – whether the search results in the target being found. One factor that profoundly affects this outcome of visual search is the *prevalence* of the target. That is, when a person is performing multiple visual search tasks in succession, such as an airport baggage security screener searching through successive x-ray images of luggage on a shift, then targets that occur infrequently – that is, are *rare* (or of ‘low prevalence’) – run a very high risk of being missed, relative to when those same targets appear in a high-prevalence context (Horowitz, 2017; Mitroff & Biggs, 2014; Wolfe et al., 2005). This is called the *low-prevalence effect*, and it has important implications for real-world visual search tasks, including airport baggage security screening and diagnostic medical imaging, as these contexts are characterized by targets that are relatively rare (e.g., weapon, tumor), but there are potentially dire consequences of missed targets. While this elevated miss rate can be studied under laboratory conditions, it is important to highlight that this effect generalizes beyond this context. Indeed, even freshly trained transportation security officers (TSOs) searching through simulated bag images have been found to succumb to inflated misses under low-prevalence conditions (Wolfe et al., 2013), as did cytologists involved in cervical cancer screening when evaluating photomicrographs of cells for abnormalities (Evans et al., 2011), and mammographers searching for cancers during the real workflow of a breast cancer-screening service (Evans et al., 2013).

Varying the prevalence of visual search has been modelled as affecting two decision criteria: (1) a person’s threshold in making the perceptual decision about target presence/absence for each part of the search array to which they attend, and (2) their quitting threshold (i.e., their threshold about when to persist with versus terminate search for that array) (Wolfe & Van Wert, 2010). Response times (RTs) on target-absent trials are said to be the key indicator of this quitting threshold (Wolfe & Van Wert, 2010). Previous research has shown that those with slower target-absent RTs miss fewer targets in low-prevalence search (Goodhew & Edwards, 2023; Peltier & Becker, 2017; Schwark et al., 2013; Thomson & Goodhew, 2021; Wolfe et al., 2005). In other words, a higher quitting thresholds (i.e., persisting longer) is associated with better performance in low-prevalence visual search relative to a lower quitting threshold.

Altered processing speed and quitting threshold have both been implicated in explaining task performance-related changes as a function of aging. Older adults have typically been found to prioritize accuracy over speed across multiple task contexts (Brébion, 2001; Forstmann et al., 2011; Hertzog et al., 1993; Vallesi et al., 2021), including high-prevalence visual search (Hommel et al., 2004; Yabuki & Goodhew, 2021). This has been attributed to both processing speed (Brébion, 2001; Forstmann et al., 2011) and strategic prioritization of accuracy over speed (Vallesi et al., 2021; Yabuki & Goodhew, 2021). In other words, older adults could respond more slowly on a variety of tasks because they require extra time to process the same amount of information, and thus it reflects an intrinsic deficit in processing speed. Alternatively, older adults could respond more slowly because they strategically prioritize accuracy over speed, without there necessarily being any intrinsic deficit. The latter is consistent with an elevated quitting threshold.

Given that older adults respond more slowly in a variety of task contexts, it is reasonable to infer that they will also respond more slowly in low-prevalence visual search. If so, there are two possible reasons for such slowing. On the one hand, it could be that older adults respond more slowly because their processing speed is slower, such that they simply need more time to process the same amount of information as a younger adult. On the other hand, it could be that older adults respond more slowly because they have an elevated quitting threshold, such that they persist longer in searching for the target before deciding it is absent. If so, then this will translate into a reduced low-prevalence effect. Here, therefore, we used RTs on low-prevalence target-absent trials to operationalize quitting threshold and RTs on high-prevalence target-present trials to operationalize processing speed, and tested whether older adults had an elevated quitting threshold or reduced processing speed, and the consequences for visual search performance.

## Method

Participants’ visual search task was to detect guns amongst arrays of photorealistic images of everyday objects. This search was performed in both low-prevalence and high-prevalence conditions (order randomized). Participants across a range of ages were recruited.

### Participants

To be able to have 95% power to replicate the magnitude of the correlation observed between another individual difference variable (i.e., cognitive failures) and low-prevalence visual search accuracy (*r*_s_ = -.26) from Experiment 1 of Goodhew and Edwards (2023) with 95% power, G*Power’s (Faul et al., 2007) point biserial correlation function indicated that N = 182 participants were required. We added 10% to 182 to account for potential exclusions (+18), and so sought to recruit N = 200. Due to a technical problem, one participant completed the study twice. Their first completion was retained, their second was replaced. At N = 200 Bayes factors for the correlation between age and LPE was in the indeterminant range in which there is not clear evidence either for the null or the alternative hypothesis, and therefore we added a further 100, but effects were still indeterminant, followed by another 100 (final N = 400), at which point key effects were out of the indeterminant range and provided definitive evidence, and therefore data collection was terminated.

We sought to recruit participants across a continuous range of ages, given that continuously sampled variables typically result in greater sensitivity than when they are dichotomized (e.g., to younger vs. older adults) (DeCoster et al., 2009). Here, therefore, we will use the terms *younger* and *older* adults in a relative sense, rather than referring to a specific prescribed age group. However, given that in our experience samples recruited from the Testable Minds platform without age restriction have a positively skewed distribution of age, such that younger adults are disproportionately sampled, we performed recruitment in six specific age bands (i.e., 20–29, 30–39, 40–49, 50–59, 60–69, 70+), where we constrained the number of participants that could sign up in each band to promote a balanced representation of ages for our continuous analysis. While there were ultimately fewer older adults available in the pool and thus were not identical numbers of participants in each age band, this approach was successful in ensuring that we maximised the range of ages in the sample (see Fig. 2). Note that these bands were not the unit of analysis – age was still treated as continuous – they were simply used to help obtain a range of ages.

All participants were recruited via the Testable Minds platform. They were required to be verified minds within the platform (as opposed to bots) including using face recognition to log in, and were paid US$15 for their time (approximately 45 min). Participants’ ages ranged between 20 and 80 years, with a mean = 42.8 years (SD = 13.9). With respect to gender, 206 reported in the free-text box ‘male’, ‘man’, or something similar or equivalent (e.g., ‘masculino’), and 173 wrote ‘female’, ‘woman’, or something similar or equivalent (e.g., ‘F’). One did not report a gender. The most frequently reported countries of birth were the UK or a country within it (N = 97), USA (N = 89), South Africa (N = 32), and India (N = 31). Three participants did not report their handedness. For the rest, most were right-handed (N = 343), whereas N = 28 were left-handed and N = 6 ambidextrous.

### Stimuli

The search arrays consisted of ten photorealistic everyday objects, which on target-present trials included a gun. These search arrays were created via custom MATLAB code that randomly selected from 64 possible images of non-gun objects (e.g., coat, doll, flute, or saucepan), plus selected one of 29 possible gun images for target-present trials. The location of the objects was randomized within the search array, and their orientation was also randomized. The size of the object images was randomly varied between 75% and 125% of the object file size. When objects overlapped, those in the foreground were made translucent to make the objects behind them visible.

**Fig. 1:**
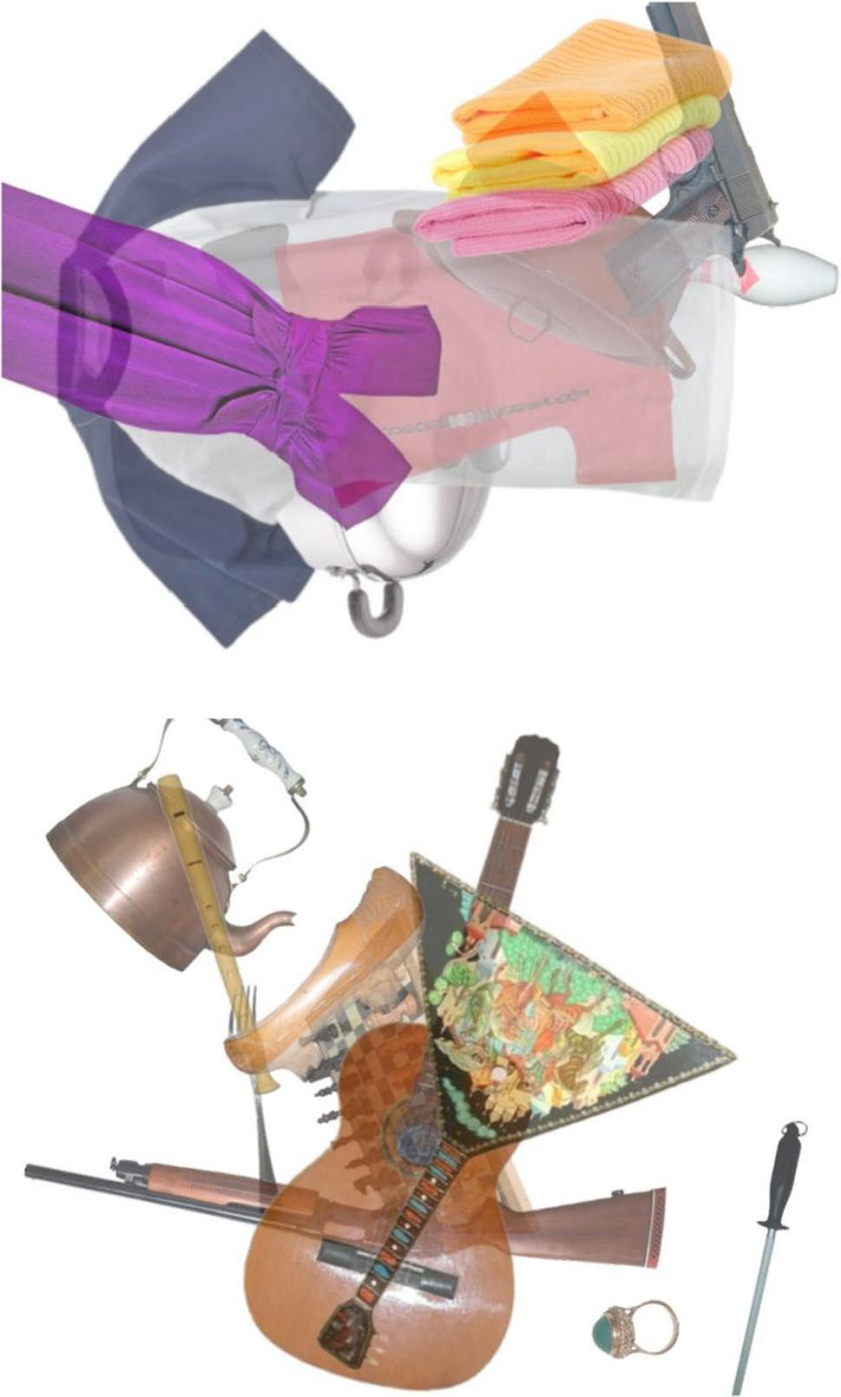
Two example target-present trials

Two experts (the two authors), one with a background in cognitive psychology and one in visual psychophysics, jointly examined all of the images produced by the code, and target-present images were replaced if they were judged too easy (i.e., gun did not overlap at all with any other objects in the array, or was very highly salient, e.g., large central object high contrast) or too difficult (i.e., gun was difficult to discern behind other objects even with prolonged inspection, or was substantially cropped by the border). All arrays were 800 × 600 pixels and contained color images on a white background. The arrays were created prior to the experiment and imported into Testable as jpegs. Two example arrays are shown in Fig. 1.

Note that the target was specifically *gun,* as opposed to the more generic category of *weapon*, because the definition of weapon is ambiguous (many items could be used as a weapon in some way, even if this is not their typical usage, e.g., a shovel). Therefore, only guns were targets. In the main experiment, there were 800 trials total, 400 per prevalence condition. In the low-prevalence condition, 16 trials contained a target (i.e., 4% target prevalence), whereas in the high-prevalence condition, 200 contained a target (i.e., 50% target prevalence). Eight practice trials preceded each block. In the low-prevalence condition, one of these was a target-present array and seven target-absent, whereas in the high-prevalence condition, four were target-present and four were target-absent.

The same 16 target-present images were used in both the low- and high-prevalence conditions to permit assessment of how prevalence, completely independent of any image effects, impacted visual search. Other images were not repeated. (In other words, the high-prevalence condition consisted of 16 of the same target-present images as the low-prevalence condition, plus 184 different target-present images.)

### Procedure

Participants completed the study online remotely via Testable (www.testable.org). Testable has been shown to have a mean precision of 3.92 ms in measuring RTs (Bridges et al., 2020). Participants first completed a Captcha, and we used Testable’s inbuilt calibration option, in which participants are instructed to resize a line shown on the screen to the size of a physical credit card, which results in the stimuli being shown at the same size on each participant’s computer on which they completed the experiment. Participants were presented with the Information Sheet, and proceeded to the experiment only if they selected the ‘Agree’ option (those who selected ‘Disagree’ did not proceed). Participants were then presented with demographic questions.

Next participants completed the visual search task. They were randomly assigned to complete either the low-prevalence or high-prevalence condition first. In the low-prevalence condition, participants received the instructions: “Here you will see an array of objects on each trial. Your task is to identify whether there is a gun amongst the objects. In this block, most of the time a gun won't be present, but sometimes it will be. If there IS a gun, press the M key. If there IS NOT a gun, press the Z key. We'll start with some practice trials”. In the high-prevalence condition, the instructions were the same except the sentence beginning *In this block* was changed to “In this block, half of the time a gun will be present and half of the time it will not be present.”

Participants then completed eight practice trials, where feedback on the accuracy of response was presented on-screen on each trial (i.e., ‘CORRECT’ or ‘INCORRECT) to help familiarize them with the task. Feedback was not provided during the experiment proper. This was done to emulate real-world visual search tasks more closely, where feedback is not provided. They were then informed that practice was complete and reminded of the task instructions before progressing to the experiment proper.

The main experiment consisted of 800 trials, 400 for each condition. Once a block was completed (i.e., either high- or low-prevalence block, depending on random assignment), the instructions for the next block appeared on screen. However, the experiment only continued when participants clicked to indicate that they read the instructions and were ready continue, and thus they could take a break between blocks they wished. The order of stimulus presentation within blocks was randomized for each participant, with the constraint in the low-prevalence condition that the first 16 trials were always target-absent. On each trial, the search array was presented until response,^1^ and there was an 800-ms intertrial interval during which the screen was blank.

### Operationalizations

Accuracy for target-present trials is equivalent to the target hit rate, or inverse of the miss rate. Similarly, accuracy for target-absent trials is equivalent to the correct rejection rate, or inverse of the false alarm rate. This means that if accuracy for target-present trials declines, then the number of misses has increased.

The low-prevalence effect (LPE) is defined as the difference in target present accuracy when the same stimuli are presented in low- versus high-prevalence contexts. To ensure that there were no perceptual differences in the images between the prevalence conditions that could contaminate this measure, we had the identical 16 search arrays appear in the Low- and High-Prevalence conditions and calculated the LPE accuracy impairment in detecting targets in them under the low- versus high-prevalence conditions (such that a positive number indicates poorer performance in the Low-Prevalence condition). We chose to focus on accuracy to calculate the LPE, rather than sensitivity (d’) within a signal detection framework, due to the false alarm rate frequently being zero in the low-prevalence condition, making the computation of d’ problematic.

Given the limited number of target-present trials intrinsic to low-prevalence designs, we did not apply RT screening at the individual trials level as this could reduce the already scarce number of target-present trials available for analysis. Instead, each participant’s *median* RT was calculated, as medians are less sensitive to outliers than means.

Quitting Threshold was operationalized via Low-Prevalence Target-Absent RT, consistent with previous research (Wolfe & Van Wert, 2010). Processing Speed was operationalized via High-Prevalence Target-Present RT (all guns). This is because target-present trials are less affected by search termination criteria than target-absent trials because search terminates when the target is found. Whereas target-present trials are infrequent under low-prevalence conditions, they are frequent under high-prevalence conditions, and therefore the time taken to search through target-present arrays under high-prevalence conditions should be most indicative of the time it takes a person to process stimuli in the array, unconfounded by targets being unexpected or absent. We chose to use all gun trials (200 trials) to increase measurement reliability compared with using only 16 trials.

Of course, neither of these operationalizations are 100% pure (e.g., processing speed will play some role on target-absent RTs in low-prevalence search), they each reflect *predominate* contributions of quitting thresholds versus processing speeds, and if both are included as predictors in the same multiple regression analysis, we can isolate the unique contribution of quitting thresholds and processing speed.

## Results

For all experiments, only data from participants who completed the experiment were analyzed. Data analysis was performed in JASP (JASP Team, 2020), except z-score screening which was done in IBM Statistical Package for the Social Sciences (SPSS).

Two participants reported an implausible age that did not match their recruitment band (126 in the 20–29 band, and 1 in the 60–69). Two participants did not report an age at all. While we know the age band of these participants and could use age substitution for them, we reasoned that (a) given the focus here on age, we wanted to be confident about participants’ actual age, (b) even if we have reasonable grounds for inferring it, failing to enter it appropriately could be considered as failing an attention check. For these reasons, we excluded these four participants from further analysis.

**Fig. 2:**
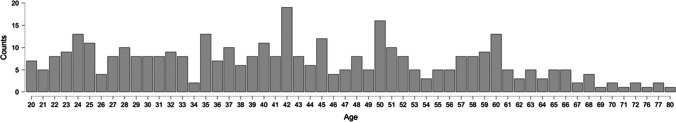
Distribution plot of participants’ age

Participants’ accuracy scores in all conditions (high- and low-prevalence, target present- and target-absent conditions, including high-prevalence target-present calculated both from the 16 arrays shared with the low-prevalence condition and calculated from all 200 target-present images) were converted to z-scores, and participants’ data was removed as outliers if the absolute value of the z-score was greater than 3.29. This led to the exclusion of sixteen cases, leaving a final sample of N = 380 for analysis. The distribution of participant age in this sample is shown in Fig. 2, which shows that we had a substantive range of ages in the sample.

Descriptive statistics for the accuracy and RT variables are shown in Table 1.

**Table 1:** Descriptive statistics for visual search accuracy and response times (RTs)

Variable	Accuracy mean (SD)	RT mean (SD)
Low-Prevalence Target-Absent	99.6% (1.4)	1,819.8ms (947.3)
Low-Prevalence Target-Present	86.1% (11.6)	1,380.9ms (562.5)
High-Prevalence Target-Absent	98.7% (2.5)	2,301.3ms (1056.9)
High-Prevalence Target-Present (16 Guns)	94.6% (6.3)	1,015.3ms (300.3)
High-Prevalence Target-Present (All Guns)	91.2% (6.3)	1,099.2ms (297.9)

16 guns = data collated from the High-Prevalence trials containing the 16 guns that were shared between the Low- and High-Prevalence conditions, whereas All Guns = data collated from the trials containing all 200 guns in the High-Prevalence condition

The RT values reflect the sample means of individual participants’ median RTs

To estimate that reliability, Cronbach’s α was computed based on the ordinal accuracy coding (0/1) for each individual trial for the 16 low-prevalence target-present trials was .57 [.51, .63], which is comparable to previous research using similar images (.59 for Thomson & Goodhew, 2021)

Data were not normally distributed, and therefore non-parametric tests were used throughout. Linear multiple regression was used (parametric), but the residuals were normal or approximately normally distributed (which is what this analysis assumes). Bayesian analyses were conducted to supplement key frequentist analyses. These were all conducted with JASP’s default priors (Ly et al., 2016).

The mean magnitude of the LPE was 8.5% (SD = 10.8). A Wilcoxon test showed that this was significantly different from zero (V = 40296, *p* < .001). Further, a Mann-Whitney U test showed that whether participants completed the low- or high-prevalence block first had no impact on the magnitude of the LPE (W = 19424.5, *p* = .131). Assignment of target keys was fixed across participants. To check that handedness did not impact the LPE, or Quitting Threshold or Processing Speed RT indices, three Mann-Whitney U-tests were conducted where handedness (dichotomised as right-handed vs. left-handed, ambidextrous and non-reports excluded) was the independent variable and LPE, Quitting Threshold and Processing Speed were the dependent variables. None produced significant effects (*p*s ≥ .300).

Do older adults have an elevated quitting threshold or slower processing speed? To assess this, a linear multiple regression analysis was conducted, where Age was the criterion, and the two predictors were Quitting Threshold and Processing Speed, which were entered simultaneously. The overall model was significant (R^2^_adj_ = .13, *p* < .001), and the regression coefficients are shown in Table 2.

**Table 2:** Regression coefficients where Age was the outcome

Predictor	Standardized regression weight (β)	Unstandardized regression weight (b)	Standard error	95% CI of b
Quitting threshold	.23***	.003	.001	.002, .005
Processing speed	.21***	.010	.002	.005, .015

***p < .001

Table 2 shows that Age was associated with elevations in both Quitting Threshold and Processing Speed. A Bayesian Linear Multiple Regression indicated that the best model was one which contained additive effects of both Quitting Threshold and Processing Speed (see Online Supplementary Material (OSM)).

Was Age associated with LPE magnitude? To assess this, we computed the Spearman’s rho correlation between Age and LPE, which was significant, *r*_*s*_(378) = -.13, *p* = .009, [-.23, -.03]. This shows that increased Age was associated with reduced LPE. A Bayesian non-parametric correlation (Kendall’s Tau = -.10, [-.16, -.03]) indicated more than three times greater evidence in favor of the alternative hypothesis that Age and LPE were associated than that they were not associated, BF_10_ = 3.47.

Does an elevated Quitting Threshold and/or slower Processing Speed *mediate* the relationship between Age and LPE? To test this, we conducted a mediation analysis, in which the Quitting Threshold and Processing Speed were tested concurrently as mediators between Age and LPE (i.e., LPE as the outcome). This revealed that Quitting Threshold was a significant negative mediator (Indirect effect Estimate = -.008, SE = .002, z = -4.72, *p* < .001, [-.012, -.005]), while Processing Speed was a significant positive mediator (Indirect effect Estimate = .006, SE = .001, z = 3.78, *p* < .001, [.003, .009]. Direct and total effects are provided in the OSM. This suggests that Quitting Threshold and Processing Speed had countervailing effects. Quitting Threshold was associated with a decreasing LPE with increasing Age whereas Processing Speed was associated with an increasing LPE with increasing Age.

This can explain why the magnitude of the age-related reduction in LPE is moderate, in contrast to the large relationship typically seen between quitting threshold and performance in younger adults (e.g., *r* = .66 correlation between target-absent RT and target-present accuracy in low-prevalence search observed in Thomson and Goodhew (2021)). It is because slower Processing Speed is a competing force undermining the benefit of older adults’ elevated Quitting Threshold.

## Discussion

Low-prevalence visual search is characterized by an inflated target miss rate relative to higher-prevalence searches. However, within younger adult samples, those who have elevated quitting thresholds (i.e., persist longer with searching each array before determining that the target is absent) perform better. Other previous research has shown that older adults respond more slowly than younger adults on a range of tasks including high-prevalence visual search. This effect has been alternatively attributed to processing speed deficits, or a strategic prioritization of accuracy over speed. Here, we had participants across a continuous range of ages perform visual search for low- and high-prevalence gun targets in an array of multiple photorealistic images of everyday objects. Older adults showed both elevated quitting thresholds and prolonged processing speed relative to younger adults. Older adults showed a reduced LPE, meaning that their miss rate was less inflated under low- compared with high-prevalence conditions. However, mediation analyses suggested that the relationship between age and LPE was mediated in different directions by quitting threshold and processing speed: quitting threshold drove a negative relationship (such that age was associated with a reduced low-prevalence detriment) while processing speed drove a positive one (such that age was associated with an exacerbated low-prevalence detriment). The implications of these findings are discussed below.

### Theoretical implications for models of aging

Aging has been theorized to be associated with declines in executive functions (De Luca et al., 2003; Fjell et al., 2016; Lacreuse et al., 2020) and inhibitory processes (Hasher & Zacks, 1988; Jurado & Rosselli, 2007; Kane et al., 1994; West & Alain, 2000). It is reasonable to infer that successful low-prevalence visual search draws on executive functions. Executive functions likely facilitate sustained attention throughout the task, persisting with unrewarding visual searches (in the sense that most do not result in a target being found), and maintaining a top-down attentional search template in the absence of regular environmental scaffolding (such as frequent exposure to the searched-for target). Similarly, inhibiting the prepotent response of target-absent when a target does finally appear likely taps into inhibitory processes. The association between individuals’ working memory capacity and performance in low-prevalence search (Schwark et al., 2013) also supports this notion that low-prevalence visual search draws upon executive and inhibition functions. Despite this, older adults did not perform worse than younger adults on the low-prevalence visual search, at least in terms of accuracy.

The absence of accuracy deficit for older adults could be seen as consistent with another school of thought espouses that if such deficits are present in aging, they are far more selective to specific processes or circumstances than previously assumed, and some cognitive processes can even improve (Rey-Mermet & Gade, 2018; Spreng & Turner, 2019; Veríssimo et al., 2022).Therefore, the finding of the present study that older adults are not impaired, could be taken to challenge the notion of wholesale decline in executive and inhibitory processes. However, given that older adults took longer to achieve this level of accuracy due at least in part to processing speed deficits, it is difficult to say that this is truly unimpaired task performance. Instead, our findings may be best explained within a compensatory framework, as explained below.

It has been debated whether the well-documented tendency for older adults to respond more slowly on a diverse array of tasks can be attributed to processing speed deficits, or a strategic prioritization of accuracy over speed. Here we found evidence of both slowed processing speed and elevated quitting thresholds (i.e., increased persistence) in older adults. This suggests rather than an either/or, both these effects occur in older adults. That is, older adults do take longer to process stimuli, but above and beyond this they are also more careful in task-related decision making.^2^

These findings can be considered to align with models of age-related compensation in cognitive function (Cabeza et al., 2018; Reuter-Lorenz & Cappell, 2008). That is, older adults may be setting a more conservative criterion in terminating search to help offset their processing speed deficits. At least in the present sample, this compensation was effective – older adults were not only not impaired, they moderately outperformed younger adults. But the complex interplay between deficits and strategies precludes a simple conclusion of age-related superiority.

### Real-World Implications for Low-Prevalence Visual Search

Here we consider the implications for personnel selection for professional visual search tasks, such as airport baggage security screening and diagnostic medical imaging. While age was associated with a reduced LPE, there are several considerations that preclude favoring older adults in personnel selection for safety-critical professional visual search tasks. First, the magnitude of this benefit was modest in size, considerably smaller than the size of the relationship between performance and other individual difference factors such as cognitive failures (Goodhew & Edwards, 2023; Thomson & Goodhew, 2021) and working memory capacity (Schwark et al., 2013). Second, while older adults did achieve this modest benefit it came at the cost of considerable task-related slowing to offset their demonstrable processing speed deficits. This may further impair the practical utility of any benefit to accuracy.

Given the clear role of quitting threshold, it may be useful to focus on promoting this to improve real-world low-prevalence visual search performance. Previous work suggests that making people respond more slowly may not be effective (Wolfe et al., 2007). This is likely because motivation is a key driver of quitting threshold, and therefore forcing slowed responses in a way that does not alter motivation will not lead to performance benefits because active searching may terminate in advance of the response. Instead, considering other individual or interventional factors that increase spontaneous quitting threshold may be more effective for improving real-world search performance.

### Generalizability

The present study recruited participants who have access to a computer and the internet and who were willing and able to complete a computerized task. This means that the older adults in the sample are likely relatively independent community-dwelling older adults. Therefore, our results likely reflect a best-case scenario for the effect of ageing and may not generalize to all older adults (e.g., those that are hospitalized or have conditions affecting their vision).

## Conclusion

Low-prevalence visual search is an important real-world task. Previous research shows elevated target misses under low- relative to high-prevalence conditions, called the LPE. Here older adults had elevated quitting thresholds, slower processing speed, and a reduced LPE. Slower processing speed exacerbated older adults’ LPE, whereas elevated quitting threshold mitigated it. These findings are consistent with compensation models of aging, and do not support preferentially selecting older adults for safety-critical search tasks.

### Supplementary information

ESM 1 (DOCX 25 kb)

## Footnotes

1 Wiegmann et al. (2006) compared discrete groups of younger and older adults when examining the effect of automation on low-prevalence visual search. These authors presented search arrays for a limited time, which limits the opportunity for older adults to excel at this task given their slower processing speed. In contrast, our design permits a more powerful test of this given that arrays were shown until response.

2 The present results are agnostic about whether the decision to be more conservative in terminating search is explicit or implicit.
