# Supplementary material for: Reduced low-prevalence visual search detriment with increasing age: Implications for cognitive theories of aging and real-world search tasks
Source: Psychon Bull Rev. 2024 Jan 30;31(4):1789–97. doi: 10.3758/s13423-024-02457-9 (PMC11358340; doi:10.3758/s13423-024-02457-9)
Supplement: Supplementary file 1 — (DOCX 25 kb) [file 13423_2024_2457_MOESM1_ESM.docx]

**Supplementary Material to accompany “Reduced low prevalence visual search detriment with increasing age: Implications for cognitive theories of aging and real-world search tasks” (Goodhew & Edwards)**

**Supplementary Item 1: Bayesian Regression Analysis where Age was the outcome (comparison to best model)**

**Bayesian Linear Regression**

| **Model Comparison - Age** | | | | | | | | | | | |
| --- | --- | --- | --- | --- | --- | --- | --- | --- | --- | --- | --- |
| **Models** | | **P(M)** | | **P(M\|data)** | | **BF _M_** | | **BF _10_** | | **R²** | |
| Quitting Threshold + Processing Speed |  | 0.333 |  | 0.997 |  | 571.802 |  | 1.000 |  | 0.136 |  |
| Quitting Threshold |  | 0.167 |  | 0.003 |  | 0.014 |  | 0.006 |  | 0.101 |  |
| Processing Speed |  | 0.167 |  | 5.962e  -4 |  | 0.003 |  | 0.001 |  | 0.093 |  |
| Null model |  | 0.333 |  | 1.618e -10 |  | 3.236e -10 |  | 1.624e -10 |  | 0.000 |  |
|  | | | | | | | | | | | |

**Supplementary Item 2: Mediation Analysis**

## Mediation Analysis

### Parameter estimates

| **Direct effects** | | | | | | | | | | | | | | | | | |
| --- | --- | --- | --- | --- | --- | --- | --- | --- | --- | --- | --- | --- | --- | --- | --- | --- | --- |
|  | | | | | | | | | | | | | | **95% Confidence Interval** | | | |
|  | |  | |  | | **Estimate** | | **Std. Error** | | **z-value** | | **p** | | **Lower** | | **Upper** | |
| Age |  | → |  | LPE |  | -0.005 |  | 0.004 |  | -1.257 |  | 0.209 |  | -0.012 |  | 0.003 |  |
|  | | | | | | | | | | | | | | | | | |
| Note.  Delta method standard errors, normal theory confidence intervals, ML estimator. | | | | | | | | | | | | | | | | | |

| **Indirect effects** | | | | | | | | | | | | | | | | | | | | | |
| --- | --- | --- | --- | --- | --- | --- | --- | --- | --- | --- | --- | --- | --- | --- | --- | --- | --- | --- | --- | --- | --- |
|  | | | | | | | | | | | | | | | | | | **95% Confidence Interval** | | | |
|  | |  | |  | |  | |  | | **Estimate** | | **Std. Error** | | **z-value** | | **p** | | **Lower** | | **Upper** | |
| Age |  | → |  | Quitting Threshold |  | → |  | LPE |  | -0.008 |  | 0.002 |  | -4.719 |  | < .001 |  | -0.012 |  | -0.005 |  |
| Age |  | → |  | Processing Speed |  | → |  | LPE |  | 0.006 |  | 0.001 |  | 3.783 |  | < .001 |  | 0.003 |  | 0.009 |  |
|  | | | | | | | | | | | | | | | | | | | | | |
| Note.  Delta method standard errors, normal theory confidence intervals, ML estimator. | | | | | | | | | | | | | | | | | | | | | |

| **Total effects** | | | | | | | | | | | | | | | | | |
| --- | --- | --- | --- | --- | --- | --- | --- | --- | --- | --- | --- | --- | --- | --- | --- | --- | --- |
|  | | | | | | | | | | | | | | **95% Confidence Interval** | | | |
|  | |  | |  | | **Estimate** | | **Std. Error** | | **z-value** | | **p** | | **Lower** | | **Upper** | |
| Age |  | → |  | LPE |  | -0.007 |  | 0.004 |  | -2.039 |  | 0.041 |  | -0.015 |  | -2.886e -4 |  |
|  | | | | | | | | | | | | | | | | | |
| Note.  Delta method standard errors, normal theory confidence intervals, ML estimator. | | | | | | | | | | | | | | | | | |

| **Total indirect effects** | | | | | | | | | | | | | | | | | |
| --- | --- | --- | --- | --- | --- | --- | --- | --- | --- | --- | --- | --- | --- | --- | --- | --- | --- |
|  | | | | | | | | | | | | | | **95% Confidence Interval** | | | |
|  | |  | |  | | **Estimate** | | **Std. Error** | | **z-value** | | **p** | | **Lower** | | **Upper** | |
| Age |  | → |  | LPE |  | -0.003 |  | 0.002 |  | -1.515 |  | 0.130 |  | -0.006 |  | 8.278e -4 |  |
|  | | | | | | | | | | | | | | | | | |
| Note.  Delta method standard errors, normal theory confidence intervals, ML estimator. | | | | | | | | | | | | | | | | | |

| **Residual covariances** | | | | | | | | | | | | | | | | | |
| --- | --- | --- | --- | --- | --- | --- | --- | --- | --- | --- | --- | --- | --- | --- | --- | --- | --- |
|  | | | | | | | | | | | | | | **95% Confidence Interval** | | | |
|  | |  | |  | | **Estimate** | | **Std. Error** | | **z-value** | | **p** | | **Lower** | | **Upper** | |
| Quitting Threshold |  | ↔ |  | Processing Speed |  | 0.336 |  | 0.049 |  | 6.817 |  | < .001 |  | 0.239 |  | 0.433 |  |
|  | | | | | | | | | | | | | | | | | |
| Note.  Delta method standard errors, normal theory confidence intervals, ML estimator. | | | | | | | | | | | | | | | | | |

- Note that absence of a significant direct effect indicates full mediation
- Note that the effects of the mediators go in opposite directions, which can account for the non-significant total indirect effects
- Note the effects were identical if variables were mean-centred
